# Patient safety culture assessment before and after safety huddle implementation*

**DOI:** 10.1590/1980-220X-REEUSP-2023-0270en

**Published:** 2024-02-12

**Authors:** Márcio Venicio Alcântara de Moraes, Ítalo Lennon Sales de Almeida, Rhanna Emanuela Fontenele Lima de Carvalho

**Affiliations:** 1 Universidade Estadual do Ceará, Fortaleza, CE, Brazil.

**Keywords:** Patient Safety, Quality of Health Care, Hospitals, Patient Care Team, Seguridad del Paciente, Calidad de la Atención de Salud, Hospitales, Grupo de Atención al Paciente, Segurança do Paciente, Qualidade da Assistência à Saúde, Hospital, Equipe de Assistência ao Paciente

## Abstract

**Objective:**

To identify whether safety huddle implementation enabled a change in patient safety culture.

**Method:**

Quasi-experimental research that assessed patient safety culture before and after safety huddle implementation.

**Results.:**

The study revealed that 53.98% completed the two safety culture assessments, with 60.1% adherence from the nursing team, with a statistically significant difference in the second assessment regarding perception of patient safety and adverse events notified (p < 0.00). Regarding good practice indicators, a statistically significant difference (p < 0.00) was observed in item 43 and improvement in almost all dimensions in the second safety culture assessment. The huddles totaled 105 days, with 100% adherence from the nursing team. Regarding checklist items, all presented satisfactory responses (above 50%).

**Conclusion:**

Safety huddles proved to be an effective tool for communication between healthcare professionals and managers, demonstrating positive impacts on good practice indicators and most safety culture dimensions.

## RESUMO

**Objetivo:**

Identificar se a implementação do *safety huddle* possibilitou mudança na cultura de segurança do paciente.

**Método:**

Pesquisa quase-experimental, que avaliou a cultura de segurança do paciente antes e após a implementação do *safety huddle*.

**Resultados:**

O estudo revelou que 53,98% preencheram as duas avaliações da cultura de segurança, com 60,1% de adesão da equipe de enfermagem, com diferença estatisticamente significativa na segunda avaliação quanto à percepção da segurança do paciente e eventos adversos notificados (p < 0,00). Quanto aos indicadores de boas práticas, observou-se diferença estatisticamente significativa (p < 0,00) no item 43 e melhoria em quase todas as dimensões na segunda avaliação da cultura de segurança. Os *huddles* totalizaram 105 dias, com 100% de adesão da equipe de enfermagem. Quanto aos itens do *checklist*, todos apresentaram respostas satisfatórias (acima de 50%).

**Conclusão:**

Os *safety huddles* revelaram-se uma ferramenta eficaz para a comunicação entre profissionais de saúde e gestores, demonstrando impactos positivos nos indicadores de boas práticas e na maioria das dimensões da cultura de segurança.

## RESUMEN

**Objetivo:**

Identificar si la implementación del safety huddle permitió un cambio en la cultura de seguridad del paciente.

**Método:**

Investigación cuasiexperimental, que evaluó la cultura de seguridad del paciente antes y después de la implementación del safety huddle.

**Resultados:**

El estudio reveló que el 53,98% completó las dos evaluaciones de la cultura de seguridad, con un 60,1% de adherencia por parte del equipo de enfermería, con diferencia estadísticamente significativa en la segunda evaluación en cuanto a la percepción de seguridad del paciente y eventos adversos reportados (p < 0,00). En cuanto a los indicadores de buenas prácticas, se observó una diferencia estadísticamente significativa (p < 0,00) en el *ítem* 43 y una mejora en casi todas las dimensiones en la segunda evaluación de la cultura de seguridad. Los huddles totalizaron 105 días, con 100% de adherencia por parte del equipo de enfermería. En cuanto a los *ítems* del checklist, todos presentaron respuestas satisfactorias (por encima del 50%).

**Conclusión:**

Los safety huddles demostraron ser una herramienta eficaz para la comunicación entre los profesionales de la salud y los gerentes, demostrando impactos positivos en los indicadores de buenas prácticas y en la mayoría de las dimensiones de la cultura de seguridad.

## INTRODUCTION

Assessing patient safety culture (PSC) at a hospital institution allows recognizing professionals’ perceptions and behaviors that influence patient safety, in addition to being an important indicator that makes it possible to understand an institution in various issues and approaches related to safe care and which behaviors and attitudes shape organizational safety culture^(1, 2)^.

It should be noted that perceptions and behaviors are individual characteristics of each professional, and may vary in different organizations or even within the same institution. PSC is understood as a product of the values, actions, conceptions, competencies and behavioral models of groups and individuals, which reflect management’s commitment to promoting a healthy and safe organization^(3)^.

Therefore, a strengthened PSC is essential, as it provides the fundamental elements for implementing safe practices to reduce adverse events^(4)^. Effective communication, for instance, is an essential tool for strengthening safety culture in healthcare institutions. It occurs when professionals receive, filter, organize and choose the appropriate channel to convey the message completely and accurately, encompassing assertive behaviors of conveying, receiving and understanding information with clarity and mutual respect, both in verbal and non-verbal communication^(5, 6)^.

In this context, the Institute for Healthcare Improvement (IHI) suggests using tools to make communication more effective, and among these tools is safety huddles^(7)^. These huddles are quick encounters with various healthcare professionals and managers, generally lasting 5 to 15 minutes, with the duration being related to the team’s needs and the nature of the activity. They follow a standard agenda with specific objectives on patient safety issues^(8)^.

Safety huddles’ effectiveness in improving care outcomes has been demonstrated in research. In one of them, it was possible to demonstrate a reduction of up to half in the mortality rate, in addition to the absence of infections related to catheters in the first two years of implementing this tool in an institution^(9)^. Another study revealed that safety huddles reduced hierarchical barriers to care, increased front-line professionals’ satisfaction and improved clinical outcomes for patients^(10)^.

Huddles are consistently related to improvements in information exchange quality, efficiency, responsibility, individual qualification, in addition to positively influencing the sense of community^(11)^. Therefore, safety huddle implementation can contribute to a culture of cooperation and partnership, promoting collective situational awareness that can lead to the elimination of harm to patients^(12)^. This sense of cooperation directs the attention of all team members to achieve a zero harm objective, resulting in greater safety and quality^(13)^.

Although the scientific literature presents considerable evidence about the effectiveness of huddles in American and European hospital environments, there is a need to assess their implementation in Brazilian institutions. Given the assumptions listed, it is considered that safety huddle implementation is relevant, as it will encourage reflection on the importance of communication for patient safety reflected in positive PSC results. It may also contribute to making the institution more reliable with favorable health indicators, allowing effective communication to be strengthened based on scientific evidence on the topic, providing decision-making, improving the care process and anticipating errors. This study was developed with the objective of identifying whether safety huddle implementation enabled changes in PSC.

## METHOD

This is quasi-experimental before-and-after research with a quantitative approach. This study reflects an intervention with safety huddle or safety huddles and PSC assessment before and after implementing this tool.

### Site

The safety huddle was implemented at a municipal hospital in northern Ceará. The institution has 119 beds dedicated to care and treatment in the specialties of medical clinic, surgical clinic, maternity, psychiatry unit, surgical center, Conventional Intermediate Care Unit (CoINCU), Type II Adult Intensive Care Unit, (Type II Adult ICU), in addition to outpatient services, pharmacy, intra-hospital and inter-hospital transport.

### Data Collection Procedure

Data collection followed three steps: 1^st^: safety culture assessment, carried out from May to June 2022; 2^nd^: safety huddle implementation (safety huddles), which lasted from August to December 2022; and 3^rd^: 2^nd^ safety culture assessment, carried out from January to March 2023.

The sample was for convenience with professionals that have worked at the hospital for a minimum of six months and working a minimum of 20 hours per week. Professionals who were away from work during the months of data collection were excluded. As a discontinuity criterion, it was considered not filling out the questionnaire in one of the stages of culture assessment.

Safety huddles were conducted by the researcher and took place in an open space common to all clinics, from August to December 2022, from Monday to Friday, in the morning, starting at 9:00 AM and lasting 20 minutes.

### Data Collection Instrument

To assess safety culture, the Brazilian version^(14)^ of the Hospital Survey on Patient Safety Culture (HSOPSC) from the Agency for Healthcare Research and Quality (AHRQ) was applied^(15)^. This questionnaire was cross-culturally adapted to Brazil in 2017 and updated in 2021^(14)^. It is an electronic system for valid, quick and reliable assessment of PSC in Brazilian hospitals^(16)^. It is known as the hospital safety culture E-questionnaire is an online, self-completed instrument and does not require an interviewer. Questions about respondents’ socio-occupational data were added to the questionnaire and it also constitutes an additional session with questions about indicators of good patient safety practices validated in the project “*Desenvolvimento e validação de indicadores de boas práticas de segurança do paciente - ISEP-Brasil*”, which enable checking the level of safety in Brazilian hospitals and specifically indicate priority problems for improvement^(17)^.

All professionals were invited to participate in the research through links sent via email. Furthermore, the researcher made daily visits to the study sectors with tablets, making them available to professionals who agreed to fill out the questionnaire at that time.

The study variables were professionals’ employment data (professional category, unit, job tenure in the hospital (in years), number of hours worked per week). Moreover, patients’ safety perception, which ranged from poor to excellent, the number of reported adverse events and good practice indicators, items 43 to 52 of HSOPSC, were considered.

Regarding safety huddles, a checklist developed by professionals from the *Hospital Geral do Grajaú* of the Sírio-Libanês Network was used, which involves questions about leadership, sizing, inputs, materials, medicines and clinical engineering^(13)^. Furthermore, the researcher added nine more questions about patient safety protocols^(18)^, namely: are there patients without identification? Safe surgery checklist is being applied. Were there any errors in the prescription, use and administration of medications? Were there any errors in the administration of blood and blood products? Have there been any patient declines? Was there an incidence of pressure injuries? Have there been healthcare-associated infections? Were there failures regarding enteral and parenteral therapies? Was there a failure in communication between professionals and health services? Four general questions were asked to involve patients and families in their own safety, totaling 23 items: were there any patient safety problems in the last 24 hours? Is there encouragement for patients and family members to participate in the care provided? Are there any factors that could put the patient at risk? Can we do anything today to protect our patients? Each checklist item was answered with “yes” or “no”, allowing participants to assign a positive or negative answer to each question.

### Study Population

To assess safety culture, the study population was 326 professionals who met the study inclusion criteria. Regarding safety huddles, they were attended by senior management or representatives, a representative of the multidisciplinary team, and a professional from each unit who was on duty, in addition to radiology technicians, administrative assistants (reception), pharmacy technicians, ambulance drivers and stretcher bearers, totaling an average of 18 professionals per day. A similar study used the same approach regarding the number of categories of participating professionals and their choice^(19)^.

### Data Analysis Procedure

Once a participant completes and submits the questionnaire, the system presents the response percentages and simple frequency of each variable in tables and graphs. *E-questionário de Cultura de Segurança Hospitalar*’s own computer program makes it possible to export data for more detailed analysis in software such as Excel.

Descriptive data analysis was carried out according to the response frequency for each item. Following the recommendation of the instrument in the Brazilian electronic version^(14)^, it was classified as strong when 75% or more of participants responded strongly agree/agree or often/always for positively formulated questions, and strongly disagree/disagree or never/rarely for negatively formulated questions. It was classified as weak when 50% or more of professionals responded negatively, choosing totally disagree/disagree or never/rarely for questions.

Regarding good practice indicators, items 43 to 52, with answers that varied (0) never, (25%) almost never, (50%) sometimes, (75%) almost always and (100%) always, all responses above 50% were considered positive. Item 50 was not included, as it refers to chemotherapy, and is not an area of activity of the assessed hospital. To compare the groups before and after, a t-test was performed in paired groups, considering p<0.05.

Safety perception ranged from poor to excellent and the number of reported adverse events was categorized. To compare these two variables between the groups of the first and second assessment, the Wilcoxon paired samples test was used, with significance considered at p<0.05.

As for the safety meeting checklist, daily monitoring occurred by completing 23 items with the option of “yes”, when it was being put into practice, or “no”, when the action was not performed. Responses were considered satisfactory when the items obtained a result above 50% in all responses.

### Ethical Aspects

The research was approved by the Research Ethics Committee of the *Universidade Estadual do Ceará* (UECE) on May 18, 2022, under Opinion 5,416,338. Ethical and legal principles were respected at all steps of the study, as provided for in Resolution 466/12 of the Brazilian National Health Council. The institution and the subjects formally authorized their participation. Professionals’ names were not identified to guarantee participants’ anonymity and obtain more reliable answers. The research complied with the recommendations of Circular Letter 1/2021-CONEP/SECNS/MoH, which provide guidelines for procedures in research with any step in a virtual environment, and the General Data Protection Law 13,709/2018, in its articles 5, 7, 11 and 13 regarding data protection by the operator and access and use of data for academic purposes.

## RESULTS

In the first and second CSP assessment, 326 questionnaires were sent and, of these, 176 (53.98%) completed both assessments. Greater participation of professionals from the nursing team (106; 60.1%), from the surgical unit (29; 16.5%) with less than a year (99; 64.7) of work in the hospital and more than 40 hours per week (100; 56.8%) stands out. Also noteworthy is the low adherence of the medical team (13; 7.4) (Table 1).

**Table 1:** Characteristics of the study sample in the two safety culture assessments (n = 176) – Sobral, Ceará, Brazil, 2023.

Variables	f (%)
**Professional category**	
Nursing technician	77(43.7)
Nurse	29(16.4)
Doctor	13(7.4)
Technician (e.g., ECG, laboratory, radiology, pharmacy)	13(7.4)
Physiotherapist, occupational therapist or speech therapist	7(4.0)
Social worker	7(4.0)
Administrative assistant/secretary	5(2.8)
Nutritionist	4(2.3)
Pharmaceutical	1(0.6)
Missing data	20(11.3)
**Unit**	
Surgery	29(16.5)
Intensive Care Unit	27(15.3)
Various hospital units/no specific unit	21(11.9)
Obstetrics	19(10.8)
Clinical medicine	18(10.2)
Others*	15(6.8)
Psychiatry/mental health	12(6.8)
Pharmacy	8(4.5)
Radiology	7(4.0)
Rehabilitation	3(1.7)
Emergency	1(0.5)
Pediatrics	1(0.5)
Missing data	15(8.5)
**Job tenure at the hospital (years)**	
Less than 1 year	99(64.7)
2 to 5 years	48(27.3)
6 to 10 years	10(5.7)
11 to 15 years	2(1.13)
16 to 20 years	1(0.5)
21 years or over	5(2.84)
Missing data	11(6.2)
**Number of hours worked per week**	
40 or more hours	100(56.8)
9 pm to 39 pm	59(33.5)
Up to 8 pm	6(3.4)
Missing data	11(6.2)

When comparing patients’ safety perception with the number of reported adverse events, a statistically significant difference was found in the second assessment (p < 0.00), with a greater preference for excellent perception. Furthermore, a statistically significant difference was observed in the number of adverse events reported by participants between the two assessments, with a decrease in the second assessment (p < 0.03) (Table 2).

**Table 2:** Comparison of patient safety perception and number of reported events. N = 176 – Sobral, Ceará, Brazil, 2023.

	1^st^ assessment	2^nd^ assessment	p
**Patient safety perception**			0,00
Poor	1(0.6)	–	–
Regular	13(7.4)	3(1.7)	
Good	108(61.4)	87(49.4)	
Great	43(24.4)	77(43.8)	
Missing data	11(6.3)	9(5.1)	
**Number of adverse events reported**			
1 to 2 cases	25(14.2)	11(6.3)	0.03
3 to 5 cases	21(11.9)	15(8.5)	
6 to 10 cases	7(4.0)	8(4.5)	
11 to 20 cases	2(1.1)	–	
More than 21 cases	1(0.6)	1(0.6)	
Total	56	35	

In relation to good practice indicators, a statistically significant difference (p < 0.00) was observed in item 43 (“When receiving verbal prescriptions about treatment...to ensure that it has been well understood?”) (Table 3).

**Table 3:** Comparison of good practice indicators before and after safety huddles – Sobral, Ceará, Brazil, 2023.

Good practice indicators	% positive responses		p
	Before	After	
	f (%)	f (%)	
43. When receiving verbal prescriptions about treatment, or any other care and procedure to be carried out with patients, does the listening professional repeat the order out loud to the person who issued it, to ensure that it has been well understood?	145(82.4)	171(97.1)	0.00
44. When receiving verbal prescriptions about treatment, care or procedure to be carried out with patients, do receiving professionals write down the order in the corresponding clinical document?	146(83)	171(97.1)	0.165
45. Before making a new prescription, do you review the list of medications that patients are taking?	147(83.6)	156(88.7)	–
46. All changes in medication are communicated clearly and quickly to all professionals involved in patient care	153(86.9)	157(89.2)	0.718
47. Is information that affects patient diagnosis communicated clearly and quickly to all professionals involved in patient care?	170(96.6)	171(97.1)	0.685
48. Before signing the informed consent, is patients or their representative asked to repeat what they understand about the possible risks of undergoing or refusing the examination, surgery or treatment involved? (Answer if you are a medical professional)	31(17.6)	20(11.4)	–
49. In patients who are likely to be terminally ill, are their preferences regarding life-sustaining measures asked in advance? (Answer only if your unit treats probably terminal patients).	20(11.3)	7(4%)	–
51. During discharge, do patients receive verbal and written instructions regarding continuity of care at home and outpatient follow-up?	162(92)	171(97.2)	0.343

**Figure 1:**
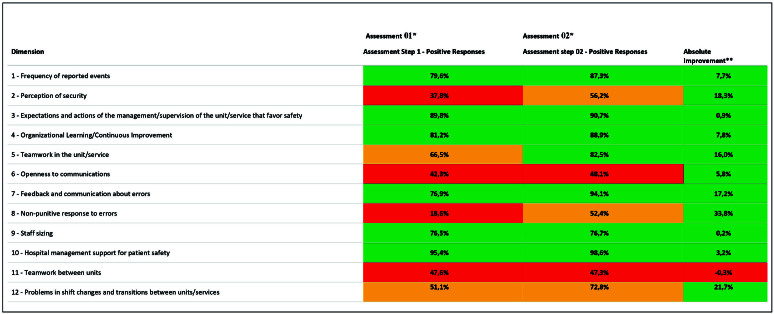
Percentage of Positive Responses by Dimension. Hospital Safety Culture E-Questionnaire.

Improvement was observed in almost all domains when comparing the scores from the 1^st^ and 2^nd^ PSC assessment. The “Non-punitive response to error” domain stands out, with an improvement of 33.8%. The “Teamwork between units” domain had a slight decrease of 0.3% (Figure 1).

**Notes:**

* Percentages of positive responses > 75% are flagged in green and percentages of positive responses < 50% are flagged in red.

** Positive improvements are flagged in green and negative improvements in red.

Safety huddles took place over five months, totaling 105 days. During the huddles, professionals from all categories participated, with the nursing team having the greatest participation on all 105 days (100%), followed by at least one representative from general management for 104 days (99%). Doctors were the professionals who participated least, with 15 days (14.2%). As for safety huddle items, all had satisfactory responses, i.e., above 50% in all responses. It is noteworthy that “Were all deliveries of materials from the pharmacy and warehouse to the units carried out on time?”, “Is the safe surgery checklist being applied?”, “Were there any errors in blood and blood product administration?” and “Can we do anything today to protect our patients?” had 100% positive responses (Table 4).

**Table 4:** Checklist items used during the 105 days of huddles – Sobral, Ceará, Brazil, 2023.

Checklist items	Yes f (%)	No f (%)
Are all leaders present at the huddle?	69(65.7)	36(34.3)
Are employee rosters covered?	90(85.7)	15(14.3)
Are there enough supplies to care for all hospitalized patients on the day?	94(89.5)	11(10.5)
Is the stock of material and medicines adequate and without risk of supply disruption?	103(98.1)	2(1.9)
Were all deliveries of materials from the pharmacy and warehouse to the units made on time?	105(100)	–
Are all equipment working properly?	98(93.3)	7(6.7)
Is there enough equipment to meet the day’s demand?	103(98.1)	2(1.9)
Will there be a need to rent or borrow equipment?	2(1.9)	103(98.1)
Does cleaning and changing linen meet bed turnover?	91(86.7)	14(13.3)
Is everyone being properly identified upon admission?	99(94.3)	6(5.7)
Have there been any patient safety issues in the last 24 hours?	25(23.8)	80(76.2)
There are patients without identification	7(6.7)	98(93.3)
Is the safe surgery checklist being applied?	105(100)	–
Were there any errors in medication prescription, use and administration?	8(7.6)	97(92.4)
Were there any errors in blood and blood product administration?	–	105(100.0)
Have there been any patient declines?	7(6.7)	98(93.3)
Was there an incidence of pressure injuries?	12(11.4)	93(88.6)
Have there been healthcare-associated infections?	10(9.5)	95(90.5)
Were there failures regarding enteral and parenteral therapies?	2(1.9)	103(98.1)
Was there a failure in communication between professionals and health services?	21(20.0)	84(80.0)
Is there encouragement for patient and family participation in the care provided?	105(100)	–
Are there any factors that could put patients at risk?	30(28.6)	75(71.5)
Can we do anything today to protect our patients?	105(100)	–

## DISCUSSION

This research aimed to identify whether safety huddle implementation enabled changes in PSC. In order to observe any effect, it was necessary to apply the safety culture questionnaire at two moments: one month before the start of the huddles and after five months.

It was observed that professionals’ adherence to the research in both assessments and safety huddles was considered satisfactory, as it obtained a return greater than 50%, in addition to the relevant participation of the nursing team in all steps. Another study found the same result regarding the participation of these professionals in culture assessment research^(16)^. Furthermore, this category is considered a profession culturally represented by women^(20)^.

The e-questionnaire authors recommend adherence of more than 50%, in addition to contraindicating assessments with samples smaller than 10 participating professionals^(14)^. Culture assessment studies had, on average, 290 participants, considering teams’ interest and concerns regarding PSC^(20, 21)^. It is worth highlighting the medical team’s low adherence at all steps of the study. Other studies obtained similar results when assessing PSC^(16)^. As for huddles, it is possible to point out similar results in the literature regarding the medical team’s low adherence, which is justified by lack of time^(10)^.

Regarding socio-labor characteristics, the results of this study are similar to those of other safety culture assessment studies^(14, 16)^. Studies found equal weekly working hours, and highlighted that long working hours can be exhausting for professionals and influence unsafe care^(16, 19)^.

Patients’ safety perception in the 2^nd^ assessment improved when compared to the 1^st^ safety culture assessment, focusing on excellent after safety huddle implementation. This health team’s conception points to a culture of safety with potential for growth and which can be encouraged when interventions that encourage communication are carried out. Research in Brazilian hospitals presented patient safety perception as fragile and growing^(20, 21)^, and that poor safety perception may be linked to lack of structures and leadership system^(14)^.

However, in this study, a statistically significant decrease was observed in the reporting of adverse events. Research that assessed PSC with 209 professionals obtained data that corroborate the findings of this study^(20)^. Authors relate low adherence to reporting adverse events with the punitive culture in healthcare organizations, making it impossible to record these occurrences that would allow organizational learning and better risk management^(16, 19, 20)^. These data contradict the percentages for the “Frequency of notified events” dimension, which had an absolute improvement of 7.7%, assuming that professionals’ perception may be more positive than the practice of reporting them. Other studies^(21,22,23)^ presented similar data, reporting that this result may be a consequence of professionals’ fear of reporting errors, lack of awareness about the importance of notification, resistance to change, lack of adequate training and work overload.

In relation to good practice indicators, items 43 to 52, growth was observed in almost all items, with a statistically significant difference (p < 0.00) in item 43 (“When receiving verbal prescriptions about treatment...to ensure that it has been well understood?”). These results are attributed to patient safety measures implemented at the hospital, points highlighted during safety huddles that, in turn, have encouraged effective communication among teams. Still for the authors of another study^(17)^, these good practice indicators contribute to facilitating the transfer of information and organizational aspects related to patient safety as well as promoting and strengthening safety culture.

Absolute improvement was observed in almost all dimensions when compared to the scores from the 1^st^ and 2^nd^ PSC assessment. The “Non-punitive response to error” dimension improved by 33.8%, however the “Teamwork between units” domain showed a slight decrease of 0.3%. When compared to other studies^(14, 16, 24)^, the results of this research were satisfactory. These results show that safety huddles can have a positive effect, since this intervention is characterized by the collective discussion of safety issues. However, teamwork between units can still be considered complex and have organizational barriers that are difficult to overcome in five months.

Regarding safety huddles, all professionals’ adherence stands out, especially the nursing team, who participated in all 105 days (100%), in addition to the participation of at least one representative of the general management in 104 days (99 %). Doctors were the professionals who participated least. For huddles to be effective, all professionals’ and senior management’s engagement is necessary, as it is a multidisciplinary and intersectoral tool capable of reducing harm to patients, providing systematic opportunities for managers, awakening a sense of responsibility and collective empowerment^(8, 11, 12)^. One of the most striking characteristics of huddles is openness to communication, as it helps interaction between sectors and resolution of safety problems, allowing a safe environment to be strengthened. Studies consider huddles to be the basis for effective communication, as they are generally interdisciplinary and strengthen partnership and/or team management^(8, 19, 25, 26)^.

The checklist used during safety huddles made huddles easier and more objective. All items received satisfactory responses (above 50%). As part of an intervention, the checklist addressed the patient safety issues described in Table 4, contributing to promoting safe care and including, in addition to the questions, confirmation from respondents whether the event happened or did not happen. Furthermore, the checklist itself served as a “means of communication”, where security information was posted. For each end of a cycle (month), the checklist allowed feedback to be provided to everyone involved regarding the problems raised during each huddle. With this, it was demonstrated to teams that the information shared in the checklist was valuable, proposing to be part of a tool or intervention that can make changes in the hospital safety culture.

A study^(27)^ considered that safety huddles must be documented, allowing the tracking of actions for identified problems, carrying out follow-up to ensure their completion. It is also necessary to create their own models to document them, such as safety checklists. They should include, in addition to the questions, the date of the huddle and confirmation from respondents whether the event happened or did not happen. The authors also state that these models contribute to measuring the effectiveness of the impact of huddles on patient safety and that consideration should be given to using safety culture surveys to check changes over time in what staff report on safety culture.

Research that has implemented safety huddles summarizes its benefits as the experience that encourages teams to think and talk about issues pertinent to safe assistance. When carried out at the beginning of shifts, they can provide good results, as they provide feedback and clarification on safety issues^(7, 8, 10, 26)^. During these safety huddles, some errors can be detected and corrected before affecting patients, as points about patient safety are discussed with multidisciplinary teams and senior management, with the possibility of reflections and improvement actions^(7–11)^.

In general, huddles made it possible to create connections with other hospital management systems so that everyone could understand each professional’s and unit’s workflow. Furthermore, for AHRQ, huddles must be adapted to teams’ needs and experience^(7)^.

The limitations of this study include the medical team’s low adherence in both safety culture assessments and safety huddles. Another limitation was the time it took to implement safety huddles and the absence of items in the checklist that reinforced the importance of reporting adverse events. It is suggested that future studies consider approaches to increase team adherence, allow more time between assessments and huddle implementation and include items related to reporting adverse events in the checklist. These measures can strengthen the validity and effectiveness of interventions in subsequent studies.

## CONCLUSION

Safety huddles proved to be an effective tool for communication between healthcare professionals and managers, playing a fundamental role in improving patient safety perception. Furthermore, its implementation demonstrated positive impacts on good practice indicators and most domains of safety culture. The results suggest that adopting safety huddles can be a valuable strategy to promote a safety culture and quality care in healthcare environments. It is recommended that these practices be incorporated as an essential part of safety protocols, aiming to improve clinical results and satisfaction of both professionals and patients.
